# Supplementary material for: Limitations in the Grain Boundary Processing of the Recycled HDDR Nd-Fe-B System
Source: Materials (Basel). 2020 Aug 10;13(16):3528. doi: 10.3390/ma13163528 (PMC7476046; doi:10.3390/ma13163528)
Supplement: Supplementary file 1 [file materials-13-03528-s001.pdf]

materials

Supplementary Materials

Limitations in the Grain Boundary Processing of the Recycled HDDR Nd-Fe-B System

Awais Ikram [1,2,3,*], Muhammad Awais [4], Richard Sheridan [4], Allan Walton [4], Spomenka Kobe [2,3], Franci Pušavec [1] and Kristina Žužek Rožman [2,3]

1 Faculty of Mechanical Engineering, University of Ljubljana, Aškerčeva cesta 6, SI-1000 Ljubljana, Slovenia; franci.pusavec@fs.uni-lj.si
2 Department for Nanostructured Materials, Jožef Stefan Institute, Jamova 39, SI-1000 Ljubljana, Slovenia; spomenka.kobe@ijs.si (S.K.); tina.zuzek@ijs.si (Z.R.)
3 Jožef Stefan International Postgraduate School, Jamova 39, SI-1000 Ljubljana, Slovenia
4 School of Metallurgy and Materials, University of Birmingham, Edgbaston, Birmingham B15 2TT, UK; m.awais@bham.ac.uk (M.A.); r.s.sheridan.1@bham.ac.uk (R.S.); a.walton@bham.ac.uk (A.W.)
* Correspondence: rana.awaisikram@yahoo.com

Received: 26 June 2020; Accepted: 5 August 2020; Published: date

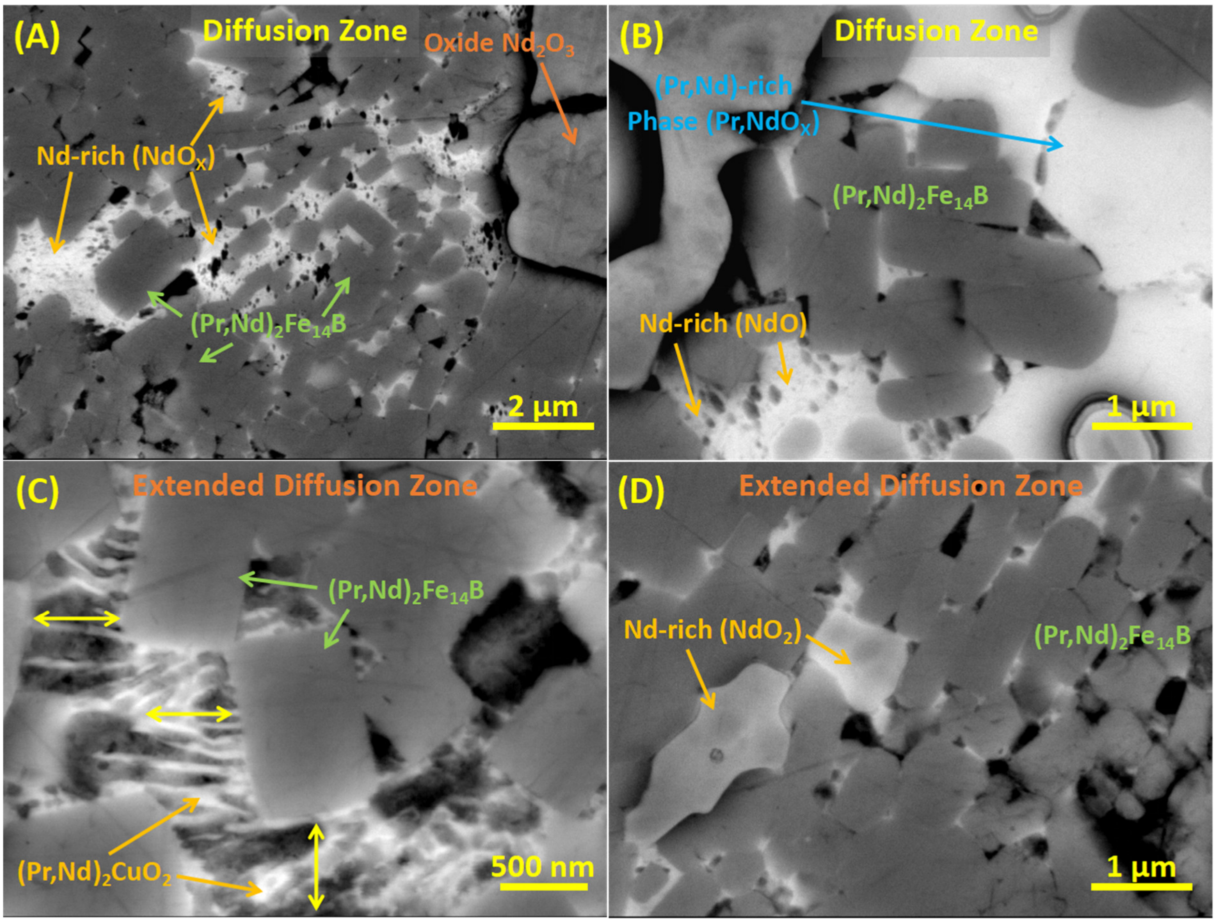

Figure S1. Supplementary backscattered electron microscopy images of (**A**) diffusion zone at 10000 X magnification showing the particle boundary on the right side and Nd-rich pool (lamellar morphology of NdO_X composition) and in the vicinity $(Pr,Nd)_2Fe_{14}B$ phases, (**B**) alternate higher magnification (20 kX) image of the diffusion zone with solidified liquid phase $(Pr,Nd)O_X$ diffused with the addition of 2 wt. % Pr-Cu alloy and the presence of Pr-containing matrix phase and lamellar

NdO_X phase (bright contrast), (**C**) high magnification (30000 X) image of diffusion zone extended beyond ~250 μm and indicating presence of complex cuprates in the thick intergranular phase, and (**D**) 20 kX magnification of the extended zone with non-uniform distribution of liquid phase along the hard matrix phase.

© 2020 by the authors. Submitted for possible open access publication under the terms and conditions of the Creative Commons Attribution (CC BY) license (http://creativecommons.org/licenses/by/4.0/).
